# SYSTEMS BIOLOGY OF HUMAN AGING - NETWORK MODEL 2023

**DOI:** 10.1093/geroni/igad104.3615

**Published:** 2023-12-21

**Authors:** John Furber

**Affiliations:** Legendary Pharmaceuticals, Gainesville, Florida, United States

## Abstract

This network is presented to aid in conceptualizing the many processes of aging, the causal chains of events, the interactions among them, feedback, and vicious cycles. It suggests promising intervention points for therapy development. The signs of human senescence are hypothesized to result from several primary causes. This network includes both intracellular and extracellular processes, ranging from molecular to whole-body. Lifestyle, environment, and proposed interventions are highlighted. Important pathways include: Nuclear mutations, transposon insertions, telomere shortening, chromosome breaks, chromatin alterations, epigenetic DNA adducts. Extracellular protein damage results in stiffness, weakness, inflammation. Altered niches for cells cause transdifferentiation, arrested cell division, cell death, cancer, stem cell depletion, tissue wasting, neurodegeneration, organ malfunction. Stiffer blood vessels promote stroke, heart disease. Lysosomes accumulate reactive, crosslinked Lipofuscin, which impairs autophagic turnover of macromolecules and organelles. Lipofuscin leaking into cytosol triggers apoptosis. Mitochondrial DNA Mutates; Lamin-A progerin, accumulates in nuclear scaffold, impairing mitosis and telomere maintenance. Nuclear Envelope Pore Proteins become oxidized, allowing inappropriate traffic of other proteins into and out of nucleus. Oxidized Aggregates in cytoplasm crosslink, resist turnover, inhibit proteasome activity, increase redox poise, and physically interfere with intracellular transport in axons. Proteasomes inhibited, reduce turnover of damaged molecules and expired molecular signals. Increased Redox Poise alters signaling and enzyme activities, and erodes telomeres. Inflammatory Cascades, promoted by damaged molecules and sick cells, further damage tissues. Neuroendocrine and immune systems degrade. Misfolded Proteins accumulate in Endoplasmic Reticulum. This diagram is maintained on the Web as a reference for researchers and students. www.LegendaryPharma.com/chartbg.html

